# Efficacy and safety of Yiqi Huoxue Yangxin decoction combined with western medicine in patients with chronic coronary heart disease: A protocol for systematic review and meta-analysis

**DOI:** 10.1097/MD.0000000000032283

**Published:** 2022-12-23

**Authors:** Bingxin Xie, Bao Wu, Mingzhang Zhao, Yili Liu, Guolei Xu, Qiao Li

**Affiliations:** a Department of Cardiovascular Medicine, Southern Branch of Guang’anmen Hospital, China Academy of Chinese Medical Sciences, Beijing, China; b Department of emergency, Southern Branch of Guang’anmen Hospital, China Academy of Chinese Medical Sciences, Beijing, China; c Department of Ultrasonography, Southern Branch of Guang’anmen Hospital, China Academy of Chinese Medical Sciences, Beijing, China; d Department of Endocrinology, Southern Branch of Guang’anmen Hospital, China Academy of Chinese Medical Sciences, Beijing, China.

**Keywords:** Coronary heart disease, meta-analysis, protocol, Yiqi Huoxue Yangxin Decoction

## Abstract

There is no evidence-based data to confirm the efficacy of Yiqi Huoxue Yangxin Decoction combined with Western medicine in patients with coronary heart disease (CHD). Therefore, in order to provide new medical evidence for clinical treatment, we used this protocol to conduct a systematic review and meta-analysis on the effectiveness and safety of Yiqi Huoxue Yangxin Decoction combined with Western medicine in patients with CHD.

**Methods:**

The systematic review protocol is registered in PROSPERO under registration number CRD42022372800. The systematic literature review will follow PRISMA guidelines (Preferred Reporting Items for Systematic Reviews and Meta-Analyses). The following search terms will be used in PUBMED, Scopus, EMBASE, Cochrane Library, CNKI, Wan Fang, and VIP on December 1, 2022. Two independent authors extract the following descriptive raw information from selected studies: study characteristics such as authors, year of publication, study design; patient demographic information such as number of patients, mean age, body mass index, and sex ratio. The primary outcome of interest is symptom scores. Secondary outcomes include ECG effective rate, improvement of blood lipid index, and adverse events. The Cochrane Bias Risk Tool is used independently by 2 reviewers to assess the risk of bias in included randomized controlled trials. The quality of retrospective studies will be assessed using the Newcastle-Ottawa scale.

**Conclusions:**

The results of our review will be reported strictly following the PRISMA criteria and the review will add to the existing literature by showing compelling evidence and improved guidance in clinic settings.

## 1. Introduction

Coronary heart disease (CHD), defined as myocardial ischemia and hypoxia caused by coronary atherosclerosis, is the leading cause of death worldwide. Globally, there are an estimated 8.92 million deaths per year due to CHD.^[1]^ The etiology of CHD is usually associated with narrowing of the luminal internal diameter of the coronary arteries and obstruction of blood circulation. Despite substantial improvements in health education in nutrition, diet, exercise, and physical activity worldwide, the morbidity, mortality, and economic burden of CHD have recently increased unevenly across geographic regions.^[2,3]^

Traditional Chinese medicine believes that CHD belongs to the category of “chest obstruction” and “heartache.” The disease lies in the heart and is related to the spleen, liver, and kidney. Qi deficiency and blood stasis are the main pathogenesis of CHD.^[4,5]^ Middle-aged and elderly people are prone to blood stasis, phlegm invasion of yang position and stagnation of qi and blood due to physical decline, resulting in obstruction of qi and blood circulation and pain due to obstruction. Therefore, patients with CHD are manifested as chest pain, shortness of breath, spontaneous sweating and palpitation.^[6–8]^ Based on this theory, the treatment of CHD in traditional Chinese medicine is mainly to supplement qi and activate blood circulation and supplement qi and nourish yin, so as to achieve the purpose of treating both symptoms and root causes. Some studies believe that the combination of supplementing qi and activating blood circulation with western medicine can significantly improve the therapeutic effect, shorten the time for symptom improvement, and have less adverse reactions and higher safety.^[9,10]^

However, there is no evidence-based data to confirm the efficacy of Yiqi Huoxue Yangxin Decoction combined with Western medicine in patients with CHD. Therefore, in order to provide new medical evidence for clinical treatment, we used this protocol to conduct a systematic review and meta-analysis on the effectiveness and safety of Yiqi Huoxue Yangxin Decoction combined with Western medicine in patients with CHD.

## 2. Materials and Methods

### 2.1. Data sources and search strategy

**Figure 1.**
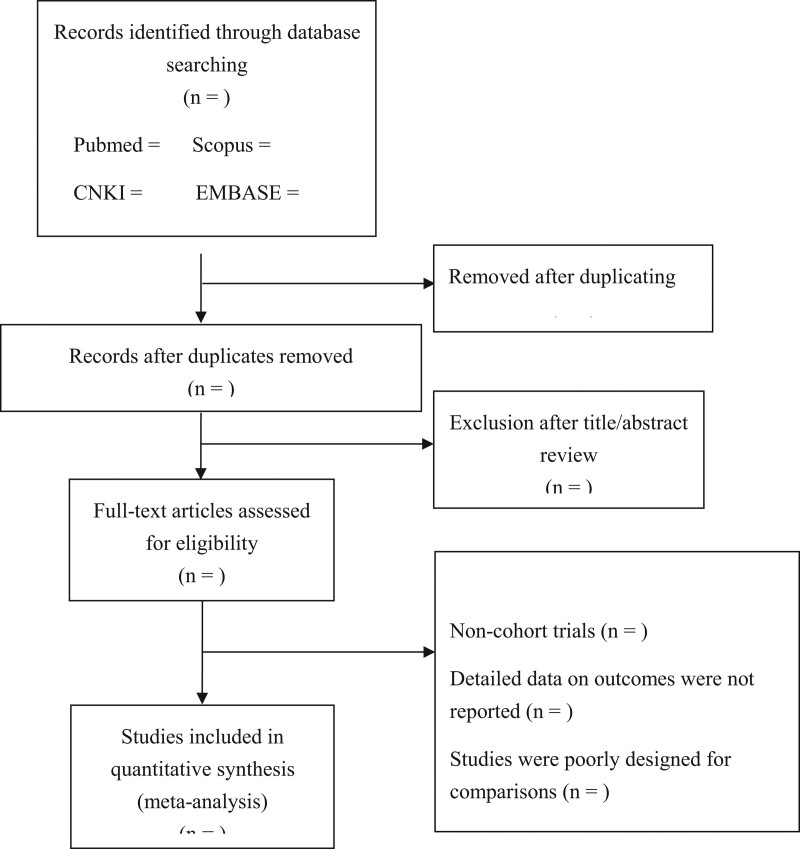
PRISMA Flow diagram describing the selection process for relevant clinical trials used in this meta-analysis.

The following search terms will be used in PUBMED, Scopus, EMBASE, Cochrane Library, CNKI, Wan Fang, and VIP on December 1, 2022, as the search algorithm: “Yiqi Yangyin” OR “Yiqi Huoxue” OR “Yiqi Yangyin Huoxue” OR “Yiqi Yangyin Jiedu” OR “tonifying qi and nourishing Yin” OR “supplementing qi and promoting blood” OR “ nourishing Yin and promoting blood” and “coronary heart disease” OR “angina pectoris.” Two searchers will draft and execute the search strategy independently, and a third member will further complete it (Fig. 1). There is no time limit on the publication date. References in the included articles will be also reviewed to identify articles that are not included in our literature search. Since this study is on the basis of published or registered studies, ethical approval and informed consent of patients are not required. The systematic review protocol is registered in PROSPERO under registration number CRD42022372800. The systematic literature review will follow PRISMA guidelines (Preferred Reporting Items for Systematic Reviews and Meta-Analyses), which include requirements considered essential for transparent reporting of results. If necessary, we will update our protocols based on any changes made throughout the course of the study.

### 2.2. Inclusion and exclusion criteria

#### 2.2..1. Inclusion criteria.

The inclusion criteria set for this paper were as follows.

Type of study: any clinical cohort study.

Type of participant: patients with chronic CHD.

Intervention group: patients treated with YHYD and western medicine.

Control group: patients treated with western medicine only.

Outcome measurements: symptom scores, ECG effective rate, improvement of blood lipid index, and adverse events.

#### 2.2..2. Excluding criteria.

The following will be excluded: noncohort trials; detailed data on outcomes were not reported or could not be calculated from the data provided; studies were poorly designed for comparisons; serial publications from the same cohort, with overlapping participants and study designs, also excluded.

### 2.3. Data extraction

Data extraction methods will follow those outlined in the Cochrane Handbook for Systematic Reviews of Interventions. Two independent authors extract the following descriptive raw information from selected studies: study characteristics such as authors, year of publication, study design; patient demographic information such as number of patients, mean age, body mass index, and sex ratio. The primary outcome of interest is symptom scores. Secondary outcomes include ECG effective rate, improvement of blood lipid index, and adverse events. If data are missing or cannot be extracted directly, we will contact the appropriate author to ensure information integration. If necessary, we will forgo extracting incomplete data.

### 2.4. Statistical analysis

Review Manager software (v 5.4; Cochrane Collaboration) is used for the meta-analysis. Continuous variables are extracted and analyzed to mean value ± SD. Standardized mean differences with a 95% confidence interval are assessed for continuous outcomes. The heterogeneity is assessed by using the *Q* test and *I*^2^ statistic. An *I*^2^ value of < 25% is chosen to represent low heterogeneity and an *I*^2^ value of > 75% to indicate high heterogeneity. All outcomes are pooled on random-effect model. A *P* value of < 0.05 is considered to be statistically significant.

### 2.5. Quality assessment

Each paper will be reviewed by one reviewer and verified by a second reviewer, and disagreements will be resolved through discussion with a third reviewer. A meta-analysis will be conducted when 3 or more trials reported an outcome of interest. Subgroup analyses are planned according to different follow-up periods and status of symptom assessment. We will also perform sensitivity analyses to assess whether differences in study design have an impact on the overall estimates and data. The Cochrane Bias Risk Tool is used independently by 2 reviewers to assess the risk of bias in included randomized controlled trials. The quality of retrospective studies will be assessed using the Newcastle-Ottawa scale. The Newcastle-Ottawa scale consists of 8 items with a total score of 9 points. “Good” is defined as an overall score of 7 to 9, “fair” as a score of 4 to 6, and “poor” as a score of less than 4.

## 3. Discussion

There is no evidence-based data to confirm the efficacy of Yiqi Huoxue Yangxin Decoction combined with Western medicine in patients with CHD. Therefore, in order to provide new medical evidence for clinical treatment, we used this protocol to conduct a systematic review and meta-analysis on the effectiveness and safety of Yiqi Huoxue Yangxin Decoction combined with Western medicine in patients with CHD.

## Author contributions

**Data curation:** Bingxin Xie, Bao Wu, Mingzhang Zhao.

**Formal analysis:** Bingxin Xie, Bao Wu, Mingzhang Zhao.

**Funding acquisition:** Qiao Li.

**Investigation:** Bingxin Xie, Bao Wu, Mingzhang Zhao.

**Methodology:** Qiao Li.

**Writing—original draft:** Bingxin Xie, Yili Liu.

**Writing—review and editing:** Guolei Xu.
